# The Sequence of Two Bacteriophages with Hypermodified Bases Reveals Novel Phage-Host Interactions

**DOI:** 10.3390/v10050217

**Published:** 2018-04-24

**Authors:** Andrew M. Kropinski, Dann Turner, John H. E. Nash, Hans-Wolfgang Ackermann, Erika J. Lingohr, Richard A. Warren, Kenneth C. Ehrlich, Melanie Ehrlich

**Affiliations:** 1 Department of Food Science, University of Guelph, Guelph, ON N1G 2W1, Canada; 2 Department of Pathobiology, Ontario Veterinary College, University of Guelph, Guelph, ON N1G 2W1, Canada; 3 Department of Applied Sciences, Faculty of Health and Applied Science, University of the West of England, Coldharbour Lane, Bristol BS16 1QY, UK; dann2.turner@uwe.ac.uk; 4 Public Health Agency of Canada, National Microbiology Laboratory, Guelph, ON N1G 3W4, Canada; john.nash@canada.ca (J.H.E.N.); Erika.Lingohr@canada.ca (E.J.L.); 5 Department of Microbiology, Immunology and Infectiology, Faculty of Medicine, Université Laval, Québec, QC G1V 4G2, Canada; ackermann4@gmail.com; 6 Department of Microbiology and Immunology, University of British Columbia, Vancouver, BC V6T 1Z3, Canada; rajw@mail.ubc.ca; 7 Hayward Genetics Center and Tulane Cancer Center, Tulane University, New Orleans, LA 70112, USA; ehrlich8@gmail.com (K.C.E.); ehrlich9@gmail.com (M.E.)

**Keywords:** bacteriophage, hypermodified bases, alpha-putrescinylthymine, ΦW-14, SP-15, *Bacillus*, Delftia, DNA sequencing, 5-hydroxymethyluracil, 5-hydroxypentyluracil

## Abstract

Bacteriophages SP-15 and ΦW-14 are members of the *Myoviridae* infecting *Bacillus subtilis* and *Delftia* (formerly *Pseudomonas*) *acidovorans*, respectively. What links them is that in both cases, approximately 50% of the thymine residues are replaced by hypermodified bases. The consequence of this is that the physico-chemical properties of the DNA are radically altered (melting temperature (Tm), buoyant density and susceptibility to restriction endonucleases). Using 454 pyrosequencing technology, we sequenced the genomes of both viruses. Phage ΦW-14 possesses a 157-kb genome (56.3% GC) specifying 236 proteins, while SP-15 is larger at 222 kb (38.6 mol % G + C) and encodes 318 proteins. In both cases, the phages can be considered genomic singletons since they do not possess BLASTn homologs. While no obvious genes were identified as being responsible for the modified base in ΦW-14, SP-15 contains a cluster of genes obviously involved in carbohydrate metabolism.

## 1. Introduction

Classically, phage DNAs were characterized biophysically on the basis of their melting temperature (Tm) and buoyant density in CsCl. These values can be correlated with the guanine plus cytosine (mol % G + C) content in the DNA [1,2,3], and a discrepancy between the values obtained with the two techniques served as a red flag for the presence of modified bases. There are a number of well-documented cases in which one of the canonical bases is completely replaced by another base (Table 1).

Partial or complete replacement of one base by another can also result in resistance to a broad range of restriction endonucleases [16,17,18], difficulty in cloning [19] and problems in dideoxy sequencing. The presence of modified and hypermodified bases in viral DNAs has been reviewed [17,20,21]. This publication will describe two phage DNAs in which thymine is only partially replaced by a fifth base.

### 1.1. Delftia Phage ΦW-14

In 1967, Andrew Kropinski using raw sewage from the Iona Island wastewater treatment plant (Richmond, BC, Canada) and *Delftia (Pseudomonas) acidovorans* Strain #14 from Roger Stanier’s culture collection at the University of California, Berkeley [22], isolated phage ΦW-14. It was named after the laboratory where it was isolated: Room 14 in the Wesbrook Building at the University of British Columbia. This virus has a head of 85 nm and a tail of 118 × 20 nm with indistinct short fibers [23] (see also [24]).

**Figure 1:**
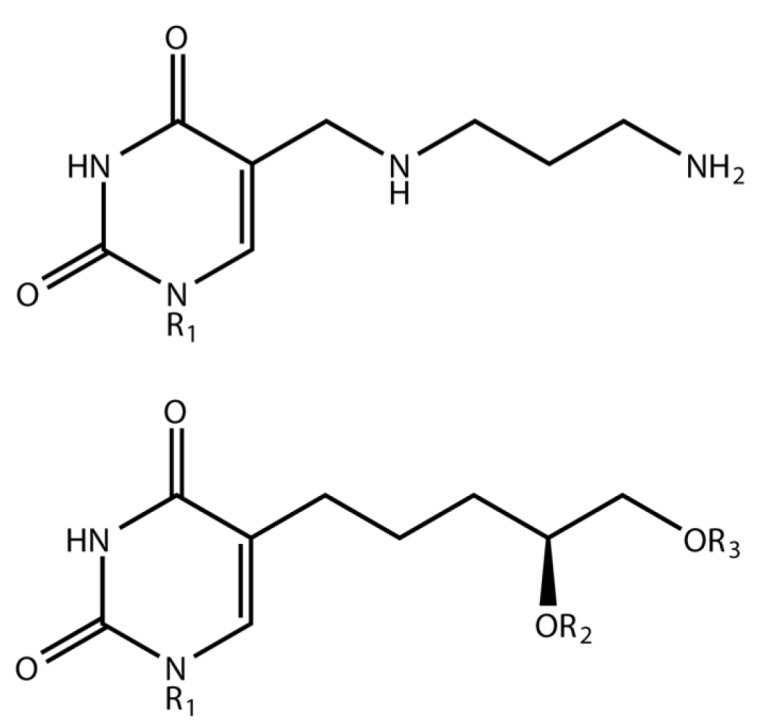
Structures of the hypermodified thymine derivatives in ΦW-14 (top) and SP-15 (bottom) phage DNAs. In both cases, R1 indicates the deoxyribosyl moiety; while in the latter case, R2/R3 represent glucosyl and phosphoglucuronolactoneresidues.

It was fully characterized with respect to its host range, adsorption rate constant (1.9 × 10^−9^ mL/min), one-step growth curve (latent period, 63 min; burst size, 300) and sensitivity to pH, temperature, sonication and UV irradiation [23]. It spontaneously generates an unusually high number of plaque morphology variants and can enter into a carrier state with its host [25]. The most exciting aspect of this research was the observation of a major discrepancy between the mol % G + C calculated on the basis of Tm measurements (71.9%) and that from CsCl buoyant density determinations (6%). Hydrolysis of the DNA with formic acid, but not perchloric acid, revealed five UV-adsorbing spots on paper chromatograms [26]. Spectrophotometric quantitation of the resolved bases indicated that the mol % G + C was in fact 54.8 and that approximately 50% of the thymine content was replaced by the fifth base, which was initially called kropinsine. The structure of this hypermodified base was elucidated through chemical analysis and NMR spectroscopy revealing it to be 5-(4-aminobutylaminomethyl)uracil, now commonly called alpha-putrescinylthymine (PutThy) [26,27] (Figure 1). 

Subsequent studies concentrated on the biosynthesis of PutThy. Phage infection caused the cessation of host DNA synthesis within 10 min, but no host genome degradation occurs. which led to the conclusion that all of the ΦW-14 nucleotide precursors are synthesized de novo [28]. Studies using a thymidine auxotroph demonstrated that the initial stage of PutThy synthesis employed a phage-specific synthase, which catalyzed the synthesis of 5-hydroxymethyl-dUMP from *N*(5),*N*(10)-methylene-tetrahydrofolate and dUMP [29]. ΦW-14-infected *Delftia acidovorans* nucleotide pools do not contain dTTP as a result of the appearance of dTTPase activity [28], but do contain hydroxymethyl dUTP (HmdUTP), resulting from the synthesis of a phage-encoded hydroxymethyluracil synthase [30].

The alpha-putrescinyl residues of ΦW-14 DNA could be labelled using ornithine [^14^C(5)], but not arginine[^14^C(U)], nor ornithine[^14^C(1)], suggesting that the putrescinyl moiety is derived from putrescine [31]. However, ornithine-labeled nucleotides were not detected in phage-infected cells [28]. The latter finding suggested that the modification occurred post-polymerization. This was investigated further using conditional lethal (amber) mutants. Several ΦW-14 mutants were affected in phage DNA synthesis [32]. For example, mutant *am37* is defective in PutThy synthesis and abnormally accumulates 5-[(hydroxymethyl)-*O*-pyrophosphoryl]uracil (HmPPUra) in the newly-synthesized phage DNA [33]. This led to the conclusion that after DNA polymerization, HmPPUra-containing DNA was modified with putrescinyl side chains to form PutThy-containing DNA. This resulted in an investigation of the origin of the putrescinyl side chains, which resulted in the finding that the *D. acidovorans* polyamines are putrescine, 2-hydroxyputrescine and spermidine [34,35]. The final stage involves the modification of a portion of the HmUra residues to form PutThy and the replacement of the remainder by Thy. The mechanisms for the latter step are unknown.

ΦW-14 DNA was found to be alkali sensitive [36]. RNA is alkali-labile because the 2′-OH on the ribose residue, under basic conditions, allows the formation of a 2′–3′ cyclic phosphate, thereby breaking the phosphodiester backbone. In ΦW-14 DNA, under basic conditions, intramolecular nucleophilic attack by the unprotonated putrescinyl amine could cleave the DNA phosphodiester backbone, whereas at neutral pH, the amine group will likely be protonated and unable to perform such a nucleophilic attack. Presumably, this can only occur because the long side chain on the thymine enables the chain to contact the phosphodiester bond. Acetylation of the PutThy residues in the DNA lowered the Tm of the DNA to that expected for its mol % G + C [37]. PutThy is required for packaging of full-length ΦW-14 DNA [24]. PutThy is not randomly distributed within the DNA (Warren, unpublished data), and its location might be important for packaging.

### 1.2. Bacillus Phage SP-15

Martha J. Taylor and Curtis B. Thorne isolated bacteriophage SP-15 from soil using *Bacillus subtilis* W-23 as the host [38]. It was characterized as an unclassified species in the family *Myoviridae* in the International Committee on Taxonomy of Viruses (ICTV) 6th Report (1995). SP-15 DNA was found to display extraordinary biochemical properties for a naturally-occurring DNA [39]. These include a unique alkaline sensitivity, reactivity with orcinol typical of a pentose, the lowest known melting temperature of any natural DNA (61.5 °C) and a high CsCl buoyant density (1.761 g/mL). Under alkaline conditions that hydrolyze RNA (0.3 M NaOH, 37 °C), but not DNA to mononucleotides, SP-15 DNA appears to become fragmented. In 1971, Julius Marmur directed his laboratory group to determine whether SP-15 DNA might be a unique example of an organism with an unusual partial DNA-RNA hybrid genome where ribose, which is orcinol reactive, would be present in addition to deoxyribose. NMR and mass spectroscopic analysis revealed that the unusual DNA modification was a hypermodified uracil with a 4,5-dihydroxypentyl group attached to the 5-position of uracil (DHPU; Figure 1 [40]). This modified uracil replaces over 50% of the normal thymine residues [41,42,43]. However, the 4,5-dihydroxypentyl modification did not explain the orcinol reactivity or the alkaline sensitivity of the DNA.

Subsequently, the alkaline sensitivity of SP-15 DNA was proposed to be due to a phosphorylated pentose of unspecified nature as part of the DHPU nucleotide residue [44]. In 1981, the phosphorylated non-backbone sugar of the SP-15 genome was identified as glucuronolactone linked via a phosphodiester group to one of the two hydroxyls of the DHPU residue (Figure 1, [45]). The second hydroxyl group is attached to a glucose residue [39,45]. It has not been determined which of the two hydroxyl groups of DHPU is glycosylated and which contains the glucuronolactone moieties. Release of the phosphoglucuronate occurs upon treatment with alkali, although the partial fragmentation of SP-15 DNA in alkali remains to be explained, as with ΦW-14 DNA, it is possible that, after alkaline cleavage of the phosphoglucuronate moiety on the DHPU, the 5-hydroxyl on the DHPU is able to intramolecularly cleave the phosphodiester backbone. This must be an infrequent occurrence, because the size of the DNA is not dramatically decreased by alkaline treatment. With phage SPO1 DNA, the side chain is not sufficiently long to allow such nucleophilic attack, and therefore, this DNA is not alkali sensitive.

Because the phosphate of the phosphoglucuronate is diesterified, it adds an additional negative charge to the hypermodified DNA residue of SP-15. Therefore, it is likely that the glucuronosyl moiety exists predominantly in its lactone form to prevent yet another additional negative charge from being present on the modified uracil. The glucose moiety is not released by alkaline hydrolysis indicating that the non-backbone phosphodiester linkage is confined to the phosphoglucuronate. The strong orcinol-reactivity of SP-15 DNA is attributable to the glucuronic acid moiety [45] rather than the previously-speculated presence of DNA backbone ribose phosphate linkages replacing some deoxyribose phosphate moieties. While evidence indicates that the DHPU structure is generated before DNA synthesis [43], it is unclear whether the addition of the glucuronic acid-1-phosphate and glucose moieties of DHPU occurs before or after the deoxynucleotide triphosphate is incorporated into the DNA during phage replication.

Lamentably, by the end of the last century, studies on these viruses came to an end because of funding problems and the lack of DNA sequence data. The hypermodified DNAs could not be readily cloned or sequenced. This changed with the introduction of clone-independent sequencing, specifically 454 pyrosequencing technology [46,47], which we used in the present study of the genomes of these extraordinary phages.

## 2. Materials and Methods

### 2.1. Host and Phages

ΦW-14 (9355-B1™) and its host *Delftia acidovorans* (den Dooren de Jong) Wen et al. (9355™) were purchased from the American Type Culture Collection (Manassas, VA, USA); while phage SP-15 (HER130) and its host *Bacillus subtilis* (HER1130) were obtained from the Félix d’Hérelle Reference Center for bacterial viruses of the Université Laval (Laval, QC, Canada).

### 2.2. Propagation

These phages were cultured in Luria broth (Difco) containing 10 mM CaCl_2_ at 30 °C and harvested post DNase I treatment by polyethylene glycol precipitation [48]. Phage DNA was isolated using the protocols described by Sambrook and Russell [49].

### 2.3. Electron Microscopy

Concentrated phage lysates were deposited on carbon-coated Formvar films on copper grids, stained with 2% uranyl acetate (pH 4.0) and examined using a Philips EM 300 electron microscope [50]. The magnification was calibrated using phage T4 tails.

### 2.4. DNA Sequencing, Sequence Assembly and Annotation

The sequence of both phages was determined using 454 technology at the McGill University and Génome Québec Innovation Centre (Montreal, QC, Canada). The sequences were assembled using Newbler and annotated using MyRAST [51,52] followed by visual inspection in Kodon 3.0 (Applied Maths, Austin, TX, USA). To eliminate the potential of terminal redundancy in these phages, the raw sequencing reads were also assembled using DNASTAR’s SeqMan NGen12 (Madison, WI, USA).

The phage proteins were scanned for homologs using the PSI-BLASTp [53], and domains and motifs were identified using the InterProScan [54] features of Geneious R7 (BioMatters Ltd., Auckland, New Zealand) and HHpred [55] in an effort to identify the functions of the proteins. In addition, the mass and pI of each of the phage proteins was recorded (Supplementary Tables S1 and S2).

Comparative genomics and proteomics: Comparative genomics was assessed using BLASTn against the non-redundant NCBI database limited to the taxonomic identifier “Viruses” (taxid: 10239), while comparative proteomics employed CoreGenes 3.5 [56,57]. Phylogenetic analysis of the major capsid and “thymidylate synthases” was conducted using “One click” mode from phylogeny.fr [58]. The data were exported in Newick format and visualized in FigTree [59].

### 2.5. GenBank

The annotated sequences of SP-15 and ΦW-14 were deposited to GenBank under the accession numbers KT624200 and GQ357915, respectively.

### 2.6. Diagrams

Schematic maps of the SP-15 and ΦW-14 genomes were prepared using the CGView Comparison Tool [60,61] and annotated with Adobe Illustrator. The chemical structures were produced using ChemDraw [62]. The short gene map diagram associated with Table 2 was prepared using EasyFig [63].

## 3. Results

### 3.1. Phage ΦW-14

**Figure 2:**
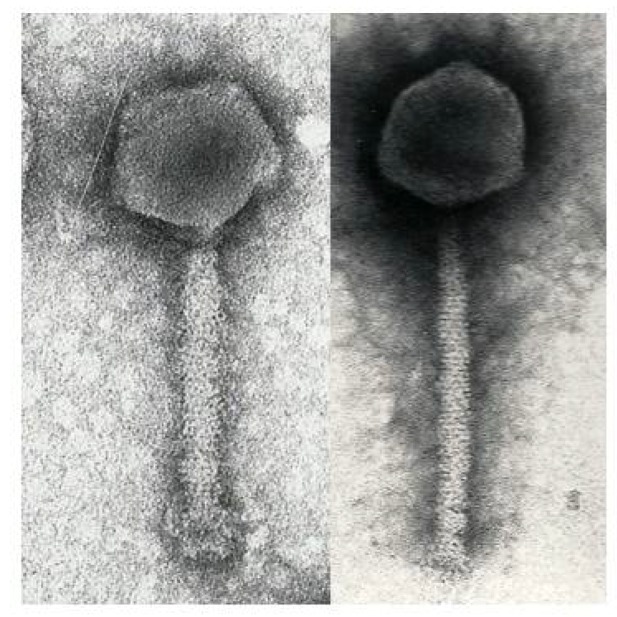
Electron micrograph of phages ΦW-14 (left) and phage SP-15 (right) stained with 2% *w*/*v* uranyl acetate.

The most recent electron micrographic analysis of this phage (Figure 2) revealed that the phage head is icosahedral as indicated by the observation of pentagonal and hexagonal particles, and the tail contractile. The diameter of the capsid is about 81 nm, while the tail is 150 nm in the extended state and 80 × 23 nm in the contracted state. A 7-nm neck, but no collar, was observed. The tail was terminated by a 33-nm baseplate to which indistinct 12-nm fibers were attached.

DNA pyrosequencing revealed a 157-kb (56.3 mol % G + C) genome with no evidence for terminal repeats and that encodes for 236 proteins and no tRNAs (Supplementary Table S1; Supplementary Figure S1). BLASTn analysis reveals that it is a genomic orphan or singleton since they do not possess BLASTn homologs [64], but it does share 47 protein homologs (16.9%) with coliphage T4, as shown using CoreGenes 3.5 [57]. Indeed, Petrov and Karam considered it part of the T4 superfamily [65].

**Figure 3:**
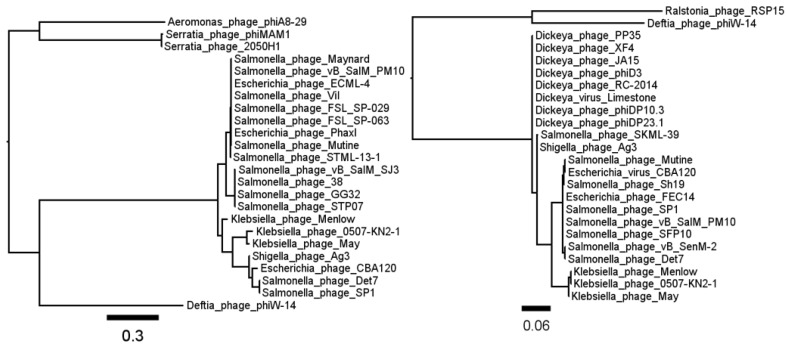
Phylogenetic analysis of the major capsid protein (gp39, left panel) and hydroxymethyluracil synthase (gp230, right panel) of *Delftia* phage ΦW-14 reveals a peripheral relationship with viruses belonging to the *Ackermannviridae* family.

As described in the Introduction, we expected to find genes that could encode a dTTPase (deoxythymidine-5′-triphosphatase), a hydroxymethyluracil synthase and possibly a novel DNA polymerase, plus enzymes involved in polyamine metabolism. The deduced 912-amino acid DNA polymerase (gp34) is related to other T4-like gp43 proteins and has its closest homologs among phages that have recently been classified to the viral family *Ackermannviridae* [66]. This is also true for the hydroxymethyluracil synthase (Figure 3). Interestingly, there is some evidence that other members of the *Ackermannviridae* family may possess modified bases derived from HmUra [67,68]. However, the only deduced ΦW-14 protein remotely related to a polyamine biosynthetic protein is a 434-amino acid polypeptide that is a putative bifunctional glutathionylspermidine synthetase/amidase (gp91). This polypeptide shows homology to proteins from three *Pseudomonas* phages-phiPMW [69], ventosus (GenBank Accession No. MG018930) and Lu11 [70,71]. Interestingly, the DNA of the latter phage is also resistant to many restriction endonucleases [72].

An important conclusion from our analysis of the genome of this virus is that the presence of a hypermodified base may not be readily assessable from the phage’s DNA sequence. Therefore, great care should be used in distinguishing amino acid sequences of hydroxymethyluridylate synthase from thymidylate synthases. Enzymatic analyses are often needed in addition to the evaluation of DNA-derived amino acid sequences when deducing the likely function of phage proteins.

### 3.2. Phage SP-15

Buoyed by our success in sequencing the ΦW-14 genome, we turned to that of SP-15. The most recent electron micrographic analysis of this phage (Figure 2) revealed it to be a large phage with an isometric head and rigid contractile tail; and the observation of occasional particles with two tails. The head diameter was 105 nm between opposite apices and the slender tail 250 × 20 nm in the extended state, showing about 56 striations with a periodicity of 3.5 nm. In the contracted state, the tail was 100 × 23 nm. Again, it showed a similarly-sized neck and no collar. The base plate was indistinct (27 × ca. 3 nm), carrying short spikes of about 10 nm in length [73].

DNA sequencing revealed that the genome was 222 kb (38.6 mol % G + C) and that it was predicted to encode 306 proteins, and no tRNAs (Supplementary Table S2; Supplementary Figure S2). It was also a genomic orphan (or singleton).

**Figure 4:**
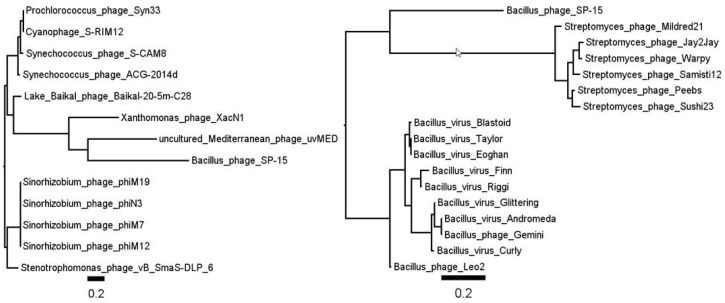
Phylogenetic analysis of the major capsid protein (gp34, left panel) and synthases (gp09, right panel) of *Bacillus* phage SP-15 reveals the former’s relationship with cyanobacterial and *Sinorhizobium* phage protein, while the gp09 synthase is related to an unclassified group of *Streptomyces* phages and *Andromedavirus*.

This phage encodes two “thymidylate synthetases”, the products of genes *9* and *130*. These two proteins both contain Pfam family *Thy1* (PF02511.9; [74]) motifs [75] and are structurally homologous to *Thermotoga maritima* flavin-dependent thymidylate synthase [76,77], though the latter protein had a low expect (E) score. The product of gene *130* has no homologs, while the gene *9* protein is peripherally related to a currently unclassified group of *Streptomyces* phages and members of the *Andromedavirus* clade. Essentially nothing is known about these phages, though their genomes have been sequenced. We hypothesize that gp9 is a thymidylate synthase, while gp130 is the hydroxymethyluridylate synthase (Figure 4).

**Figure 5:**
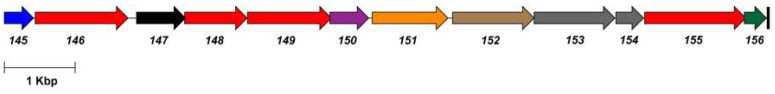
Gene map of the 10.8-kb region of SP-15 that encodes numerous host-related genes involved in carbohydrate biosynthesis (in red). Color code: blue, ssDNA binding protein; black, HNH homing endonuclease; purple, diguanylate cyclase; brown, teichoic acid biosynthetic protein; grey, hypothetical proteins; and green, thioredoxin. The terminal black box corresponds to a rho-independent terminator.

**Figure 6:**
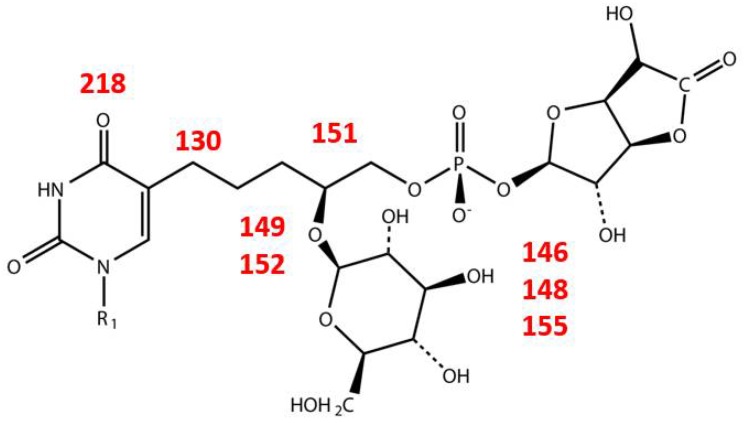
Detailed structure of the hypermodified base in SP-15 DNA with potential phage proteins indicated in red.

DNA replication involves a phage DNA polymerase, which is the product of genes *109* and *111,* resulting in the incorporation of dTMP and DHPU into the nascent DNA. The biosynthesis of the DHPU, its glucuronolactone-1-phosphate and glucosyl moieties probably involves enzymes specified by genes within a 10-gene cluster (genes 146 to 155) in the SP-15 genome. These are listed in Table 2 and Figure 5 and integrated into an overall picture in Figure 6.

Most of the cluster genes are not present in the genomes of other bacteriophages that are known to contain modified uracil moieties. The phage gene encoding CDP-glycerol glycerophosphotransferase (also called teichoic acid synthase, gene 151) together with uridylyl transferase (gene 148) and a glucose dehydrogenase (gene 146) may be required for synthesis of a phospho-esterified dihydroxypentyl chain, which could then be transferred to the uracil by a UTP-glucose-1-phosphate uridylyltransferase encoded by another gene in the cluster. The UDP-glucose dehydrogenase and several other genes in the cluster are similar to some genes in *Bacillus* and *Micrococcus* species known to catalyze teichoic and teichuronic acid biosynthesis [78,79]. In *Micrococcus luteus*, a 440-kDa enzyme complex (encoded by a gene cluster like that of SP-15) contains two types of glycosyltransferases, a glucuronosyltransferase and other enzymes that are necessary for teichuronic acid biosynthesis [79].

Horizontal gene transfer leading to mosaic phage genomes is frequently raised in manuscripts (see, e.g., [80]), but the origins of the nonhomologous regions are frequently not addressed. Here, we have a clear example where the closest homologs are to be found in non-prophage regions of bacterial genomes. As such, our results clearly rank with the discovery of host photosynthesis genes in cyanomyoviruses [81] and quorum sensing genes in *Clostridioides difficile* phages [82].

Whether or not insertion of the (modified) DHPU or the normal thymine deoxynucleoside triphosphate precursors occurs randomly in the DNA has not been determined. Analysis of di-, tri-and tetranucleotides in SP-15 DNA digests revealed that adjacent DHPU residues are not found in the DNA backbone [45]. This suggests that the phosphodiester within the modified uracil base is preventing, by either steric or charge interference, the positioning of two neighboring DHPU bases during replication. The biological functionality of SP-15′s hypermodified DNA is still unclear, but the modified base does not seem to be simply protecting the phage DNA from phage-encoded DNA degradative enzymes and thereby allowing only degradation of host DNA [43]. DNA containing normal amounts of DHPU is apparently not needed for phage gene expression, but proper phage packaging does appear to require the presence of the hypermodified DHPU [83].

To the next generation of phage scientists goes the challenge of identifying other novel nucleotides in phage DNA. Microchemical analyses and single-molecule real-time (SMRT) DNA sequencing using PacBio or Nanopore instruments [84] should assist in meeting this challenge. In the case of these two phages with baroque DNAs, much more research is needed on the biochemistry and genetics of the post-polymerization reactions in ΦW-14, and specifically what controls the insertion of side chains. For phage SP-15, studies are needed on what controls the differential incorporation of dTTP and dDHpentylUTP; and where the two saccharides are attached. Both phages are unusually sensitive to strand scission caused by alkali, yet this has not been mechanistically investigated.

## Figures and Tables

**Table 1:** Examples of bacteriophages where a canonical base is completely replaced by another base.

Phage	Host	Substitution	Reference
ΦR1-37	*Yersinia*	Thy → Ura	[4]
PBS1	*Bacillus*	Thy → Ura	[5]
SPO1, SP8, SP10	*Bacillus*	Thy → 5HmUra (a portion becomes α-glutamylthymine)	[6,7,8,9]
XP-12	*Xanthomonas*	Cyt → 5MeCyt	[10,11]
Teven phages	*Escherichia*	Cyt → 5HmCyt (glycosylated)	[12,13]
RL38JI	*Rhizobium*	Cyt → 5HmCyt (variably glycosylated)	[14]
S-2L	*Synechococcus*	Ade → 2AminoAde	[15]

Thy, thymine; Ura, uracil; 5HmUra, 5-hydroxymethyluracil; Cyt, cytosine; 5MeCyt, 5-methylcytosine; 5HmCyt, 5-hydroxymethylcytosine; Ade, adenine; 2AminoAde, 2-aminoadenine.

**Table 2:** Summary of the genes thought to be involved in the modification of the 4,5-dihydroxypentyl group attached to the 5-position of uracil (DHPU) residues in SP-15 DNA.

Gene	Product	Function
*11*	glucose-6-phosphate isomerase	Glc-6-P → Fru-6-P
*129*	acyl carrier protein reductase	
*146*	UDP-glucose dehydrogenase	UDP-Glc → UDP-GlcA
*148*	UTP-glucose-1-phosphate uridylyltransferase	Glc-1-P → UDP-Glc
*149*	glycosyl transferase	
*151*	CDP-glycerol:poly(glycero-phosphate) glycerophosphotransferase	
*152*	glycosyl transferase	
*155*	phosphomannomutase	Glc-6-P → Glc-1-P
*219*	dCMP deaminase	dCMP → dUMP
*130*	hydroxymethyldeoxyuridine synthase	dUMP → dHmdUMP

## Supplementary Materials

The following are available online at http://www.mdpi.com/1999-4915/10/5/217/s1, Figure S1: *Delftia* phage phiW-14 genomic map, Table S1: *Delftia* phage phiW-14 genome features, Figure S2: *Bacillus* phage SP-15 genomic map, Table S2: *Bacillus* phage SP-15 genome features.
