# Supplementary figures and images for: The Sequence of Two Bacteriophages with Hypermodified Bases Reveals Novel Phage-Host Interactions
Source: Viruses. 2018 Apr 24;10(5):217. doi: 10.3390/v10050217 (PMC5977210; doi:10.3390/v10050217)

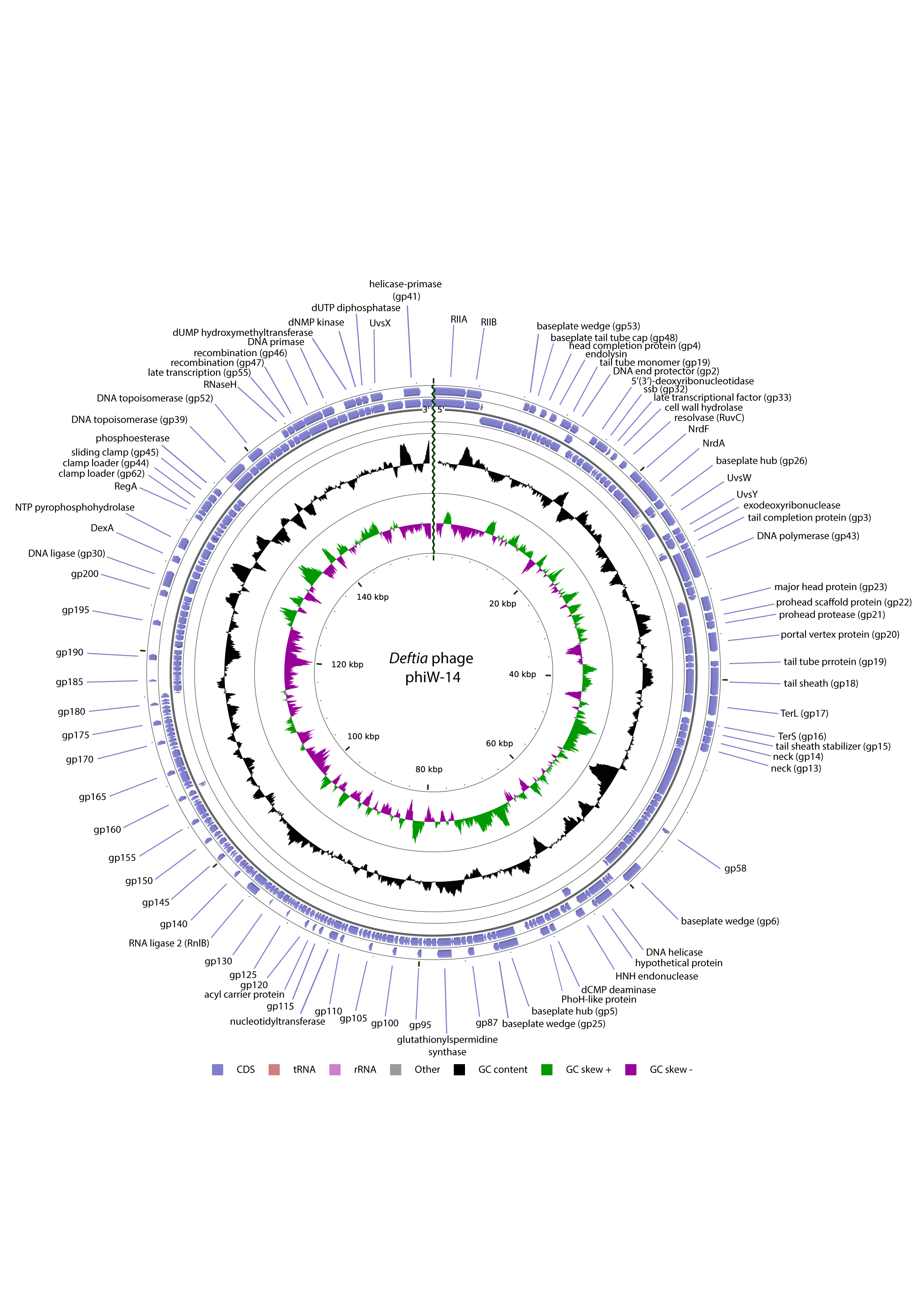

Supplement: Supplementary file 1 [file viruses-10-00217-s001.zip › Figure S1 Delftia phage phiW-14 genomic map.tif]

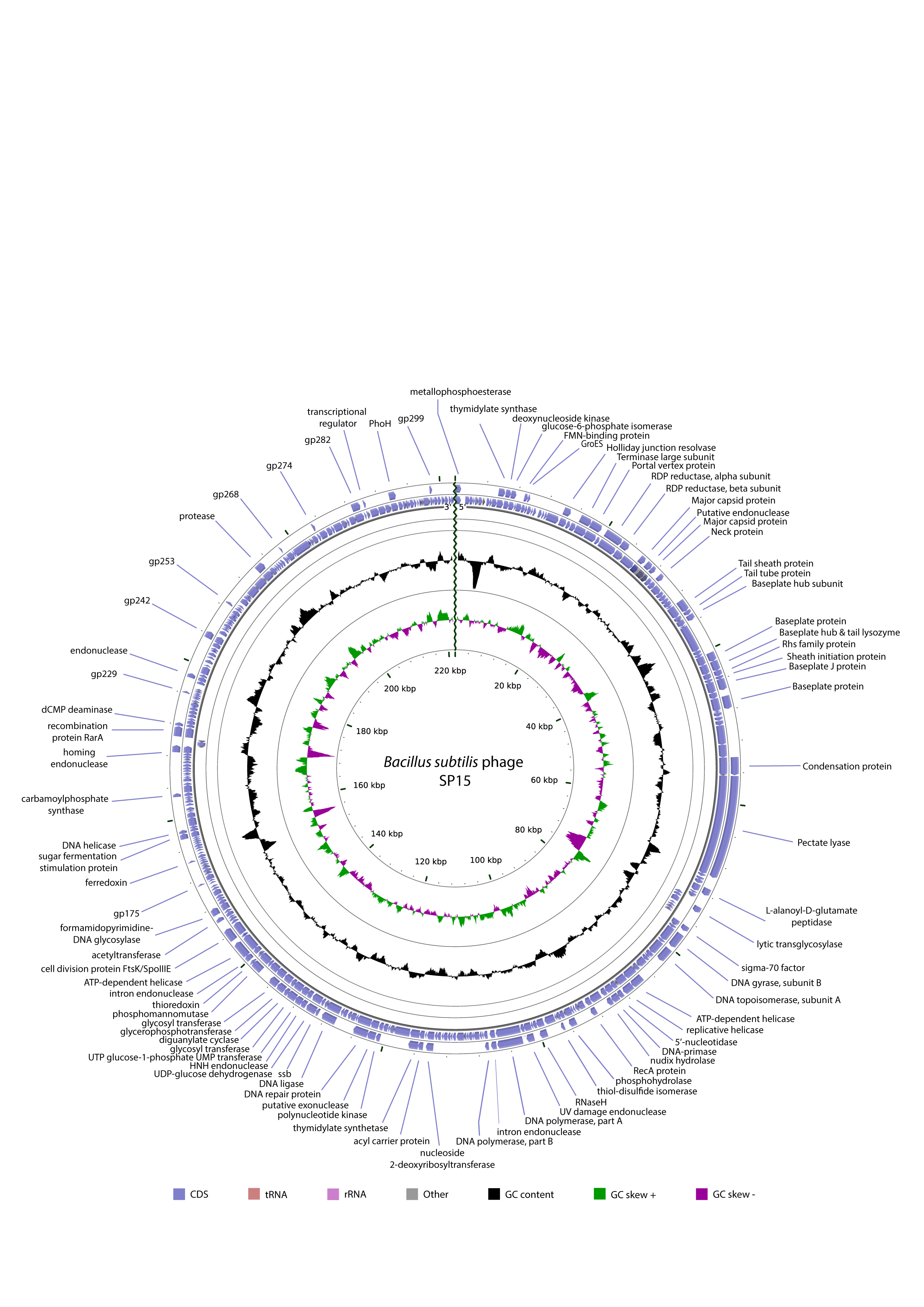

Supplement: Supplementary file 1 [file viruses-10-00217-s001.zip › Figure S2 Bacillus phage SP-15 genomic map.tif]
